# Impairment of Neuronal Mitochondrial Quality Control in Prion-Induced Neurodegeneration

**DOI:** 10.3390/cells11172744

**Published:** 2022-09-02

**Authors:** Mo-Jong Kim, Hee-Jun Kim, Byungki Jang, Hyun-Ji Kim, Mohd Najib Mostafa, Seok-Joo Park, Yong-Sun Kim, Eun-Kyoung Choi

**Affiliations:** 1 Department of Biomedical Gerontology, Graduate School of Hallym University, Chuncheon 24252, Korea; 2 Ilsong Institute of Life Science, Hallym University, Seoul 07247, Korea; 3 Hongcheon Institute of Medicinal Herb, Hongcheon 25142, Korea; 4 Department of Microbiology, College of Medicine, Hallym University, Chuncheon 24252, Korea

**Keywords:** prion disease, dynamin-related protein 1, mitochondrial quality control, mitochondrial fission, mitophagy, neurodegeneration

## Abstract

Mitochondrial dynamics continually maintain cell survival and bioenergetics through mitochondrial quality control processes (fission, fusion, and mitophagy). Aberrant mitochondrial quality control has been implicated in the pathogenic mechanism of various human diseases, including cancer, cardiac dysfunction, and neurological disorders, such as Alzheimer’s disease, Parkinson’s disease, and prion disease. However, the mitochondrial dysfunction-mediated neuropathological mechanisms in prion disease are still uncertain. Here, we used both in vitro and in vivo scrapie-infected models to investigate the involvement of mitochondrial quality control in prion pathogenesis. We found that scrapie infection led to the induction of mitochondrial reactive oxygen species (mtROS) and the loss of mitochondrial membrane potential (ΔΨm), resulting in enhanced phosphorylation of dynamin-related protein 1 (Drp1) at Ser616 and its subsequent translocation to the mitochondria, which was followed by excessive mitophagy. We also confirmed decreased expression levels of mitochondrial oxidative phosphorylation (OXPHOS) complexes and reduced ATP production by scrapie infection. In addition, scrapie-infection-induced aberrant mitochondrial fission and mitophagy led to increased apoptotic signaling, as evidenced by caspase 3 activation and poly (ADP-ribose) polymerase cleavage. These results suggest that scrapie infection induced mitochondrial dysfunction via impaired mitochondrial quality control processes followed by neuronal cell death, which may have an important role in the neuropathogenesis of prion diseases.

## 1. Introduction

Prion diseases are characterized by the accumulation of beta-sheet-enriched prion protein (PrP^Sc^), which is converted from normal cellular prion protein (PrP^C^), excessive glial cell activation, neuronal cell death, and spongiform degeneration [1,2]. These features indicate that prion infection leads to neurological symptoms such as dysfunctions in memory, movement, and cognition [3]. It has been reported that mitochondrial dysfunction via endoplasmic reticulum (ER) stress, endothelial nitric oxide synthase (eNOS), and oxidative stress, which results in reduced ATP production, increased mitochondrial reactive oxygen species (mtROS) production, and neurodegeneration [4,5,6,7]. In addition, it has been suggested that aberrant mitochondrial quality control induces neuronal cell death in both in vitro and in vivo models of various neurodegenerative diseases, including Alzheimer’s disease (AD), amyotrophic lateral sclerosis (ALS), Huntington’s disease (HD), Parkinson’s disease (PD), and prion diseases. Previous studies have revealed that excessive mitochondrial fission and mitophagy in neuronal cells are increased in AD and HD patients compared to healthy adults and have been implicated in the neuropathological mechanisms of both diseases [8,9].

Mitochondria are key organelles in energy metabolism and are involved in a multitude of cellular events, such as calcium homeostasis, ROS production and apoptosis. To maintain mitochondrial function, several pathways of mitochondrial quality control have evolved, such as mitochondrial dynamics and mitophagy [10]. Mitochondrial dynamics are regulated by large guanosine triphosphatases (GTPases) in the dynamin family, which are involved in the fission and fusion of the two lipid bilayers that surround mitochondria. In particular, mitochondrial fission is mediated by the recruitment of cytosolic dynamin-related protein 1 (Drp1) to mitochondria, forming spirals that severely constrict both the inner and outer mitochondrial membranes [11]. Mitochondrial fission is modulated by accessory proteins, including mitochondrial fission 1 (Fis1), mitochondrial fission factor (Mff), and mitochondrial dynamics proteins of 49 and 51 kDa [12]. In addition, several studies have shown that Drp1 recruitment to mitochondria is regulated by posttranslational modifications, including phosphorylation [13]. More specifically, mitochondrial dynamics are involved in the response to infection, stress, changes in nutrient supply, or cellular physiology and play an important role in apoptosis [14,15,16].

Mitophagy is a selective lysosomal degradation pathway for the elimination of damaged mitochondria or misfolded protein aggregates in mitochondria and plays an important role in the physiology and pathophysiology of all cell types [17]. Recently, it has been reported that mitophagy plays a critical role in neuronal function and survival by maintaining healthy mitochondria and inhibiting neuronal cell death [18]. Moreover, mitophagy is tightly regulated in response to alterations in cellular physiology, such as stress and infection, and plays a critical role in the progression of neuropathogenesis via processes such as astrogliosis and neuronal cell death in neurodegenerative diseases [19,20].

Mitochondrial quality control has been implicated in brain homeostasis, which involves an interconnected network between neurons and glial cells. Moreover, impairment of mitochondrial quality control was shown to contribute to neuropathogenesis via the induction of glial cell activation and neuronal inflammation. In addition, it has been reported that prion infection is involved in aberrant mitochondrial quality control and altered energy-metabolism-related protein expression in various brain regions of scrapie-infected mice and sporadic Creutzfeldt–Jakob disease (sCJD) patients [21,22,23]. However, the mitochondrial quality-control-mediated neuropathological mechanisms in prion diseases are still unclear.

In this study, we investigated the relationship between mitochondrial quality control and neuropathological mechanisms in prion diseases. We demonstrated that scrapie infection induced aberrant mitochondrial fission and mitophagy triggered robust neuronal cell apoptosis and impaired mitochondrial function, resulting in the induction of neuropathological changes in prion diseases.

## 2. Materials and Methods

### 2.1. Human and Animal Models

Normal and sCJD brain tissues were obtained from the Biosafety Level-III Autopsy Center for CJD (Hallym University Sacred Heart Hospital, Anyang, Korea). The pathologic features of the sCJD patients are described in Table 1. C57BL/6J mice (4–6 weeks old) were originally purchased from the Jackson Laboratory (Bar Harbor, ME, USA) and housed in specific pathogen-free conditions in the animal facility of the Ilsong Institute of Life Science (Seoul, Korea) for the development of animal models of prion disease. An original stock of the 22L scrapie strain was kindly provided by Dr. Alan Dickinson of the Agriculture and Food Research Council and the Medical Research Council Institute (Neuropathogenesis Unit, Edinburgh, UK). For scrapie infection, the mice were intracerebrally inoculated with 30 µL of 1% (*w*/*v*) brain homogenate in phosphate-buffered saline (PBS, pH 7.4) prepared from 22L-injected mice or from control mice that had been injected with normal brain homogenate. Prion diseases were diagnosed according to clinical criteria, including ataxia, stiff tail, and hind leg paresis. The incubation period was calculated as the interval from the day of inoculation to the day of terminal clinical disease. The mice were euthanized at the onset of the terminal stage (150 ± 10 days postinfection (dpi)).

Animal and human experiments were approved by the Institutional Animal Care and Use Committee (Seoul, Korea) or Institutional Review Board of Hallym University (Chuncheon, Korea) (HMC 2020-0-0310-03, HIRB-2021-003).

### 2.2. Primary Cortical Neuronal (CxN) Cell Cultures and Scrapie Infection

Primary cortical neuronal (CxN) cells were cultured from 15-day-old embryos of pregnant C57BL/6J mice and cultured as previously described [24]. Briefly, embryonic brains were dissected, stripped of the meninges, and minced with forceps. The minced tissue was incubated in 0.125% trypsin (Gibco, Grand Island, NY, USA) and 2 mg/mL DNase I (Sigma–Aldrich, St Louis, MO, USA) in phosphate-buffered saline (PBS) at 37 °C for 15 min. Cells were dissociated by pipetting, cultured on poly-D-lysine (PDL)-coated chamber slides (Nunc Lab-Tek II, Invitrogen, Carlsbad, CA, USA) with neurobasal medium containing B-27 Plus supplement (Gibco) and incubated at 37 °C in 5% under CO_2_. After 2 days, the CxN cells were infected with 0.01% 22L scrapie-infected brain homogenate (22L) and normal brain homogenate (CON) in PBS. At 4 dpi, the medium was removed from the CxN cells, and the cells were washed twice in fresh culture medium. The medium was then changed every week.

### 2.3. Western Blot Analysis

Cells and brain tissues from the control and 22L scrapie-infected mice were homogenized with modified radioimmunoprecipitation assay (RIPA) buffer (50 mM Tris-HCl, pH 7.5, 150 mM NaCl, 1 mM EDTA, 1% Nonidet P-40, 0.5% sodium deoxycholic acid, and 0.1% sodium dodecyl sulfate) and a protease inhibitor (Pierce Biotechnology, Rockford, IL, USA). The protein concentration was determined using a BCA assay kit (Pierce Biotechnology). Protein samples (40 μg/lane) were subjected to sodium dodecyl sulfate-polyacrylamide gel electrophoresis (SDS–PAGE) with a NuPAGE 4–12% Bis-Tris SDS–PAGE (Invitrogen) or 4–15% Criterion TGX precast midi protein gel (Bio–Rad, Hercules, CA, USA) and transferred to a polyvinylidene difluoride (PVDF) membrane (Millipore, Billerica, MA, USA) using an electrotransfer system (Bio–Rad). The membrane was then blocked with 5% nonfat dry milk in PBST (8 mM Na_2_HPO_4_, 2 mM KH_2_PO_4_, 138 mM NaCl, 2.7 mM KCl, pH 7.4, and 0.1% Tween 20) for 1 h at room temperature (RT) and probed with primary antibodies against mouse monoclonal anti-Drp1 (1:2000, BD Transduction Laboratories, San Diego, CA, USA), mouse monoclonal anti-TOM20 (1:5000, Abcam, Cambridge, MA, USA), mouse monoclonal anti-OXPHOS (1:1000, Abcam), mouse monoclonal anti-HSP90 (1:5000, BD Transduction Laboratories), rabbit monoclonal anti-cytochrome c (1:1000, Cell Signaling Technology, Danvers, MA, USA), rabbit monoclonal anti-cleaved poly (ADP-ribose) polymerase (PARP) (1:1000, Cell Signaling Technology), rabbit polyclonal anti-phospho-Drp1 Ser 616 (1:1000, Cell Signaling Technology), rabbit polyclonal anti-cleaved caspase-3 (1:1000, Cell Signaling Technology), and rabbit polyclonal anti-GAPDH (1:5000, Abcam) in PBST overnight at 4 °C. The membranes were incubated with the appropriate secondary antibodies (1:5000, Enzo, Farmingdale, NY, USA) conjugated to horseradish peroxidase (HRP) for 2 h at RT. For the detection of PrP^Sc^, samples were digested with 20 μg/mL proteinase-K (PK) for 30 min at 37 °C and then incubated with mouse monoclonal anti-PrP (1:5000, 3F10) antibody [25]. Bound antibodies were visualized using a chemiluminescent substrate (ATTO, Tokyo, Japan) and imaged with an ImageQuant^TM^ LAS 4000 apparatus (GE Healthcare Life Sciences, Piscataway, NJ, USA).

### 2.4. Subcellular Fractionation

To isolate the cytosolic and mitochondrial fractions from mouse and human brain tissues, the samples were homogenized, and then the cytosolic and mitochondrial fractions were isolated as previously described [26]. Briefly, mouse brain tissues were lysed in cold isotonic homogenization buffer (10 mM Tris-HCl, pH 7.4, 250 mM sucrose, 10 mM KCI, 1.5 mM MgCI_2_, and 1 mM EDTA with a protease inhibitor cocktail tablet. Briefly, the lysates were centrifuged at 800× *g* for 5 min at 4 °C, and then the supernatant was centrifuged at 13,500× *g* for 15 min at 4 °C to obtain a crude mitochondrial pellet. The crude mitochondrial fraction was resuspended for washing and centrifuged at 13,500× *g* for 15 min at 4 °C. For the primary cortical neuronal cells, the cytosolic and mitochondrial fractions were extracted by using a mitochondria isolation kit for cultured cells (Thermo Fisher Scientific, Rockford, IL, USA).

### 2.5. Immunohistochemistry and Immunofluorescence

Neutral-buffered formalin-fixed mouse brains were cut into 6 μm-thick slices, and the sections were used for immunohistochemical staining of P-Drp1 as previously described [27]. Additional brain sections were deparaffinized in xylene, hydrated in a graded ethanol series and then subjected to antigen retrieval with citrate buffer at pH 6.0. The sections were then incubated with 5% normal goat serum (Jackson ImmunoResearch, West Grove, PA, USA) and then incubated with mouse monoclonal anti-HSP60 (1:500, Invitrogen) and rabbit polyclonal anti-phospho-Drp1 S616 (1:200, Cell Signaling Technology), rabbit polyclonal anti-LAMP1 (1:200, Abcam), and rabbit monoclonal anti-LC3B (1:200, Cell Signaling Technology) antibodies in PBST overnight at 4 °C. After primary antibody incubation, the sections were incubated with Alexa Fluor 488 goat anti-mouse IgG and 568 goat anti-rabbit IgG (1:1000, Invitrogen) antibodies. Images were obtained using an LSM700 confocal scanning microscope (Carl Zeiss, Oberkochen, Germany). For immunofluorescence staining, cells were fixed with 4% paraformaldehyde (PFA) for 10 min. For detection of PrP^Sc^, the cells were exposed to 98% formic acid for 7 min at RT after fixation and before permeabilization. Cells were incubated with mouse monoclonal anti-TOM20 (1:500, Abcam), rabbit polyclonal anti-phospho-Drp1 S616 (1:200, Cell Signaling Technology), rabbit polyclonal anti-LAMP1 (1:200, Abcam), rabbit monoclonal anti-LC3B (Cell Signaling Technology), or goat polyclonal anti-PrP (1:200, Santa Cruz Biotechnology, Santa Cruz, CA, USA) antibodies in PBST overnight at 4 °C. Cells were subsequently incubated with either Alexa Fluor 488- or 647-conjugated goat anti-mouse IgG, 568-conjugated goat anti-rabbit IgG, or 488-conjugated donkey anti-goat IgG (1:1000, Invitrogen), and then cells were mounted in 4′,6-diamidino-2-phenylindole (DAPI)-containing Vectashield antifade mounting medium (Vector Laboratories, Burlingame, CA, USA) to label the nuclei, which were visualized using an LSM700 confocal laser scanning microscope.

### 2.6. Image Acquisition and Quantification

Images were obtained using a Zeiss LSM 700 confocal solid-state laser scanning microscope (405 nm 5 mW, 488 nm 10 mW, 555 nm 10 mW, and 639 nm 5 mW) equipped with either a Zeiss 20×/numerical aperture (NA) 1.2 or 40×/NA 1.2 water immersion objective lens, with sequential-acquisition setting. Images were captured at a resolution of 1024 × 1024 pixels.

For colocalization analysis, we used the EzColocalization plugin of ImageJ (NIH) software version 1.53a (Bethesda, MD, USA) to determine the Pearson coefficient per image or per region of interest as previously reported [28,29]. Briefly, image files were imported into ImageJ/Fiji (NIH) software version 2.3.0 (Bethesda, MD, USA) and split into separate channels. The two channel images including signals corresponding to the target proteins were input into EzColocalization plugin. The Pearson’s correlation coefficient (PCC) and Manders’s 1 (M1) and 2 (M2) were quantified using all pixels or Costes’ automatic threshold pixels, respectively [30]. For quantification, in CxN cells, 5–10 cells from each group with at least 5 randomly selected fields were analyzed. Data were obtained from at least six independent experiments. In human brain sections, the frontal lobe of the human brains from 3 CJD patients or 1 non-CJD patient with at least 9 randomly selected fields were examined for colocalization.

### 2.7. Cell Viability Assay

Cells were plated in a PDL-coated 96-well chamber slide and then exposed to brain homogenates. At 21 dpi, the cells were incubated with culture medium containing the proliferation reagent Cell Counting Kit-8 proliferation reagent (CCK8; Dojindo Molecular Technologies, Kumamoto, Japan) in a humidified 5% CO_2_/95% air incubator for 1 h at 37 °C. Subsequently, the absorbance at 450 nm was measured using an enzyme-linked immunosorbent assay reader (VersaMax; Molecular Devices, Sunnyvale, CA, USA).

### 2.8. DNA Construction

The p-mito-RFP-EGFP plasmid was generated as previously described with minor modifications [31]. Briefly, p-RFP-EGFP with the LC3 coding sequence removed from ptfLC3 was a gift from Tamotsu Yoshimori (Addgene plasmid #21074; http://n2t.net/addgene:21074 (accessed on 8 January 2020); RRID: Addgene_21074). The mitochondrial targeting signal sequence of human cytochrome c oxidase subunit VIII amplified in pMitotimer was a gift from Zhen Yan (Addgene plasmid #52659; http://n2t.net/addgene:52659 (accessed on 8 January 2020); RRID: Addgene_52659) and was inserted at the N-terminus in the p-RFP-EGFP frame.

### 2.9. Mitophagy Assay

Mitophagy assays were performed as previously reported [32,33]. Briefly, cells were plated in a PDL-coated 4-well chamber slide and then exposed to brain homogenates. At 6 dpi, transfections were carried out with Lipofectamine 2000 according to the manufacturer’s instructions (Thermo Fisher Scientific). For Mtphagy Dye staining, cells were plated in a PDL-coated 4- or 96-well plate or chamber slide and then exposed to brain homogenates. Cells were cultured until 21 dpi and incubated with culture medium containing 100 nM Mtphagy Dye (Dojindo Molecular Technologies) in a humidified 5% CO_2_/95% air incubator for 30 min at 37 °C. Mitophagy and Mtphagy Dye evaluation were performed using an LSM700 confocal solid-state laser scanning microscope 20×/NA 1.2 or 40×/NA 1.2 water immersion objective lens with sequential-acquisition setting. ImageJ/Fiji software was used to quantify the fluorescence intensity, indicative of Mtphagy Dye, of a total number of 5–10 cells for each group in at least 5 random fields and at least nine independent experiments.

### 2.10. Measurement of Mitochondrial Membrane Potential (ΔΨm) and mtROS

The ΔΨm was analyzed with JC-1 staining (Invitrogen) according to the manufacturer’s recommendations. Cells were plated in PDL-coated 4- or 96-well plates or chamber slides and then exposed to brain homogenates. The cells were cultured until 21 dpi, and then the cells were incubated with culture medium containing 2 μM JC-1 reagent in a humidified 5% CO_2_/95% air incubator for 15 min at 37 °C and analyzed using a fluorescence plate reader (Infinite F200, TECAN, Männedorf, Switzerland). To measure mtROS, cells were incubated with HBSS containing 10 μM MitoSOX^TM^ Red reagent (Invitrogen) in a humidified 5% CO_2_/95% air incubator for 15 min at 37 °C according to the manufacturer’s recommendations. mtROS evaluation was performed using an LSM700 confocal solid-state laser scanning microscope 20×/NA 1.2 or 40×/NA 1.2 water immersion objective lens with sequential acquisition setting. ImageJ/Fiji (NIH) software version 2.3.0 was used to quantify the fluorescence intensity, indicative of mtROS, of a total number of 100 cells for each group in at least 5 random fields and at least nine independent experiments.

### 2.11. Determination of Glycolysis and ATP Levels

To evaluate ATP levels, a total ATP assay using the luciferase-based ATP Enzylight Kit (BioAssay Systems, Hayward, CA, USA) was applied according to the manufacturer’s instructions as previously described [32]. Glycolysis was estimated by determining the lactate levels in the conditioned medium using the Lactate-Glo Assay Kit (Promega, Madison, WI, USA) according to the manufacturer’s instructions and a fluorescence plate reader (Infinite F200).

### 2.12. Quantitative Real-Time Reverse Transcription Polymerase Chain Reaction (qRT-PCR)

Total cellular RNA extraction, complementary RNA synthesis, and qRT-PCR were performed as previously reported [34]. Briefly, total cellular RNA was extracted from the cells and brain tissues using an RNA purification Kit (GeneAll, Seoul, Korea) and subsequently quantified by qRT-PCR using a SYBR Green PCR master mix kit (Thermo Fisher Scientific) according to the manufacturer’s instructions. The quantification of mitochondrial DNA copy number by mitochondrial DNA (mtDNA) to nuclear DNA (nucDNA) ratio was determined by qRT-PCR as described previously [35]. qRT-PCR was performed using specific primers for Drp1, actin, mtDNA (NADH dehydrogenase, MT-ND2), and nucDNA (platelet/endothelial cell adhesion molecule 1, Pecam 1), as listed in Table 2. Data were normalized to actin for Drp1 and nucDNA for mtDNA.

### 2.13. Measurement of Mitochondrial Length

Quantification of the mitochondrial length was performed in TOM20 fluorescence as previously reported [36]. Briefly, the cells were plated in PDL-coated 4-well chamber slides and then exposed to brain homogenates. At 21 dpi cultures, cells were fixed with 4% PFA for 10 min. To detect PrP^Sc^, cells were exposed to 98% formic acid for 7 min at RT after fixation and before permeabilization. Cells were incubated with mouse monoclonal anti-TOM20 (1:500, Abcam) and goat polyclonal anti-PrP (1:200, Santa Cruz Biotechnology) in PBST overnight at 4 °C. The cells were subsequently incubated with either Alexa Fluor 488 donkey anti-goat IgG or Alexa Fluor 568 goat anti-mouse IgG (1:1000, Invitrogen) for 1 h at RT. Cells were visualized with a 20×/NA 1.2 or 40×/NA 1.2 water immersion objective lens on a Zeiss LSM 700 confocal solid-state laser scanning microscope, using 488 nm excitation for PrP^Sc^, 555 nm for TOM20, and 405 for DAPI. Image post-acquisition processing was performed with algorithm (Momito; www.uqtr.ca/LaboMarcGermain) (accessed on 4 July 2022) [37] and ImageJ/Fiji software. Briefly, the “skeletonize” plugin was used to define the structural elements (mitochondrial tubule, end, or junction) of the mitochondrial network. The mitochondrial network obtained after skeletonization of TOM20-labeled mitochondria was used to measure mitochondria length with Momito as described previously [38,39,40]. For quantification, 5–10 cells for each group with at least 3 randomly selected fields were analyzed. Data were obtained from at least nine independent experiments.

### 2.14. Caspase Activity Assay

Caspase-3/7 activity was determined using the Caspase-Glo 3/7 Assay Kit (Promega) according to the manufacturer’s instructions. Briefly, the cells were plated in a PDL-coated 96-well plate, incubated with Caspase-Glo 3/7 reagent for 1 h at RT and then analyzed using a fluorescence plate reader (Infinite F200).

### 2.15. Statistical Analysis

Statistical analyses were performed by GraphPad Prism 9 program (GraphPad Software, San Diego, CA, USA). Differences between the groups were compared by two-tailed unpaired Student’s *t*-tests or one-way analysis of variance (ANOVA) followed by Tukey’s post hoc test. The data are expressed as mean ± SEM of at least three independent experiments. Statistical significance was reached at *p* < 0.05.

## 3. Results

### 3.1. Scrapie Infection Induces Mitochondrial ROS (mtROS) Production and Reduces Mitochondrial Membrane Potential (ΔΨm)

**Figure 1:**
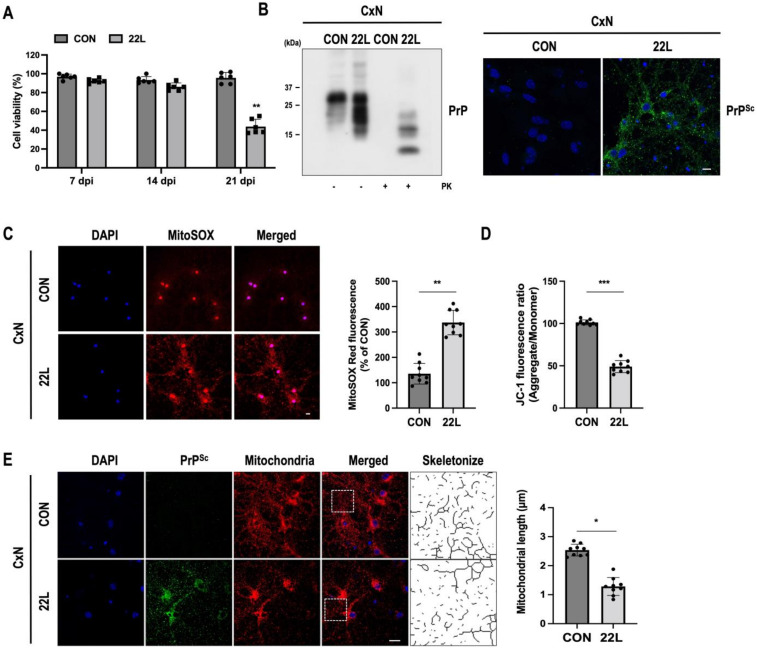
Scrapie infection induces mitochondrial ROS (mtROS) production and the loss of the mitochondrial membrane potential (ΔΨm) followed by mitochondrial fragmentation. (**A**) The relative cell viability of primary cortical neuronal (CxN) cells infected with either normal brain homogenate (CON) or 22L scrapie-infected brain homogenate (22L) for 7, 14, and 21 days post infection (dpi). Representative data is relative to controls of each time point, respectively, and analyzed using CCK8 reagent (*n* = 6, ** *p* < 0.01; two-sided Student’s *t*-test). (**B**) Deposition of prion protein (PrP) in control (CON) and 22L scrapie-infected (22L) CxN cells at 21 dpi. PrP was detected using an anti-PrP (3F10) antibody by Western blot analysis in the presence or absence of proteinase K (PK) (20 μg/mL). Using confocal microscopy, PrP^Sc^ was detected with an anti-PrP (C-20) antibody after treatment with 98% formic acid (right panel). Scale bar, 20 μm. (**C**) Detection of mitochondrial ROS (mtROS) by MitoSOX staining (red) using confocal microscopy in control (CON) and 22L scrapie-infected (22L) CxN cells at 21 dpi. The intensities of the mtROS fluorescence were measured and quantified for each group (*n* = 9, ** *p* < 0.01; two-sided Student’s *t*-test). (**D**) The ΔΨm was evaluated with JC-1 staining using a fluorescence reader (*n* = 9, *** *p* < 0.001; two-sided Student’s *t*-test). (**E**) Quantification of mitochondrial length with staining for the mitochondrial-specific marker TOM20 (red) and PrP^Sc^ (green) under confocal microscopy, and the morphological skeleton was analyzed with algorithm (Momito; *n* = 9, * *p* < 0.01; two-sided Student’s *t*-test). PrP^Sc^ (green) was detected with an anti-PrP (C-20) antibody after treatment with 98% formic acid. Nuclei were stained with DAPI (blue); *n* = number of independent experiments. Enlarged insets (dashed white line boxes) showed the sections at a higher magnification, scale bar, 20 μm. Bullets in bar graph represent datasets from independent experiments.

Scrapie infection induces a variety of cellular stresses and alters calcium homeostasis, which causes mitochondrial dysfunction and damage [41,42,43]. First, we found that the viability of CxN cells infected with mouse-adapted scrapie strain 22L was gradually decreased in a time-dependent manner and significantly decreased at 21 dpi (Figure 1A). The accumulation of PK-resistant PrP^Sc^ was also confirmed by Western blot analysis and fluorescent staining (Figure 1B). To examine the mtROS content and mitochondrial membrane potential (ΔΨm) changes after scrapie infection, both control and 22L scrapie-infected CxN cells were stained with MitoSOX and JC-1, respectively. As shown in Figure 1C,D, significantly increased mtROS generation and decreased ΔΨm were observed in infected CxN cells compared to control cells. Next, we measured mitochondrial length to determine whether scrapie infection affects the dynamics of mitochondrial morphology in CxN cells. As shown in Figure 1E, the mitochondrial length of infected cells was significantly reduced by 50% compared with that of control cells, which showed more fragmented mitochondria. Taken together, these results suggest that scrapie infection leads to an increase in mtROS generation and a decrease in ΔΨm followed by dynamic changes in mitochondrial morphology and fragmentation of the mitochondrial network.

### 3.2. Scrapie Infection Regulates Drp1-Mediated Mitochondrial Fission

**Figure 2:**
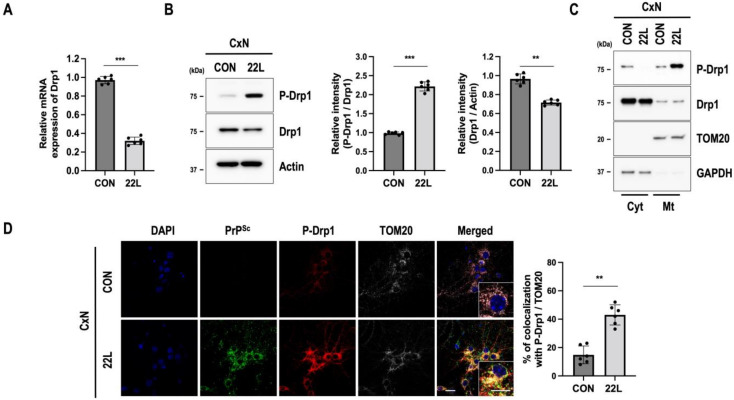
Scrapie infection induces phosphorylation of Drp1 at Ser616 (P-Drp1), which colocalizes with PrP^Sc^ in the mitochondria. (**A**) The mRNA level of Drp1 was analyzed using qRT-PCR (*n* = 6, *** *p* < 0.001; two-sided Student’s *t*-test). (**B**) Phosphorylation of Drp1 at Ser616 (P-Drp1) in control (CON) or 22L scrapie-infected (22L) CxN cells at 21 dpi was examined by Western blotting. The intensities of the bands were measured and quantified for each group (*n* = 6, *** *p* < 0.001, ** *p* < 0.01; two-sided Student’s *t*-test). (**C**) Drp1 and P-Drp1 levels in the cytosolic (Cyt) and mitochondrial (Mt) fractions. TOM20 and GAPDH were used as markers of the mitochondrial and cytosolic fractions, respectively. (**D**) The triple-localization of PrP^Sc^ (green) with P-Drp1 (red) and TOM20 (white) was determined using confocal microscopy. Quantification of mitochondrial fission by P-Drp1 and TOM20 and mitochondrial translocation was analyzed using EzColocalization plugin for ImageJ software version 1.53a (*n* = 6, ** *p* < 0.01; two-sided Student’s *t*-test). DAPI (blue) was used to counterstain the nuclei; *n* = number of independent experiments. Enlarged insets (white line boxes) showed the sections at a higher magnification, scale bar, 20 μm. Bullets in bar graph represent datasets from independent experiments.

Mitochondrial fission relies on the recruitment of Drp1 from the cytoplasm to the mitochondrial outer membrane. The phosphorylation of Drp1 at serine 616 (P-Drp1) regulates its localization and induces mitochondrial fission [12]. Since we found changes in mitochondrial morphology and fragmentation, the expression and localization of the mitochondrial fission protein Drp1 in control and 22L scrapie-infected CxN cells were evaluated. We found that both the mRNA and protein levels of Drp1 were decreased in total lysates of 22L scrapie-infected CxN cells, whereas P-Drp1 was increased in both total lysates and the mitochondrial fractions of 22L scrapie-infected CxN cells compared to those of control cells (Figure 2A–C). Next, to determine whether P-Drp1 is localized to mitochondria, we carried out immunofluorescent staining with P-Drp1 and TOM20 (a mitochondrial marker). As shown in Figure 2D, a strongly increased P-Drp1 signal was colocalized with TOM20-positive mitochondria in PrP^Sc^-positive 22L scrapie-infected CxN cells.

To support these changes in the mitochondria of 22L scrapie-infected CxN cells, we also examined Drp1 expression and its localization in the brains of 22L scrapie-infected mice. Similar to the results obtained from in vitro experiments, decreased Drp1 and significantly increased P-Drp1 were observed in whole-brain homogenates and mitochondrial fractions from 22L scrapie-infected brains (Supplementary Figure S1A–C). Moreover, intense immunoreactivity of P-Drp1 was observed in the cerebral cortex of scrapie-infected mice compared with that of control mice (Supplementary Figure S1D). In addition, upregulated P-Drp1 was mainly colocalized with the mitochondrial marker protein HSP60 (Supplementary Figure S1E).

It has been reported that scrapie infection regulates Ras homolog family member A/Rho-associated protein kinase (RhoA/ROCK) activity through the interaction of PrP^Sc^ with RhoA and p190RhoGAP in 22L scrapie-infected hippocampal neuronal cells [44] and that RhoA/ROCK activity modulates Drp1-mediated mitochondrial fission [45]. Thus, to elucidate whether RhoA/ROCK activity is involved in mitochondrial fission in 22L scrapie-infected CxN cells, cells were treated with either Y27632 (a ROCK inhibitor) or Tat-C3 (a specific inhibitor of RhoA) after scrapie infection. As shown in Supplementary Figure S2, interestingly, inhibition of RhoA and Rock led to decreases in both Drp1 phosphorylation and Drp1 localization to the mitochondria that were accompanied by decreased mtROS and effectively restored ΔΨm (Supplementary Figure S3). These findings indicate that scrapie-infection-mediated RhoA/ROCK activity is involved in Drp1-mediated mitochondrial fission by regulating Drp1 phosphorylation and its mitochondrial localization, which affects cell survival by restoring ΔΨm and increasing cell viability.

### 3.3. Scrapie Infection Promotes Mitophagy

**Figure 3:**
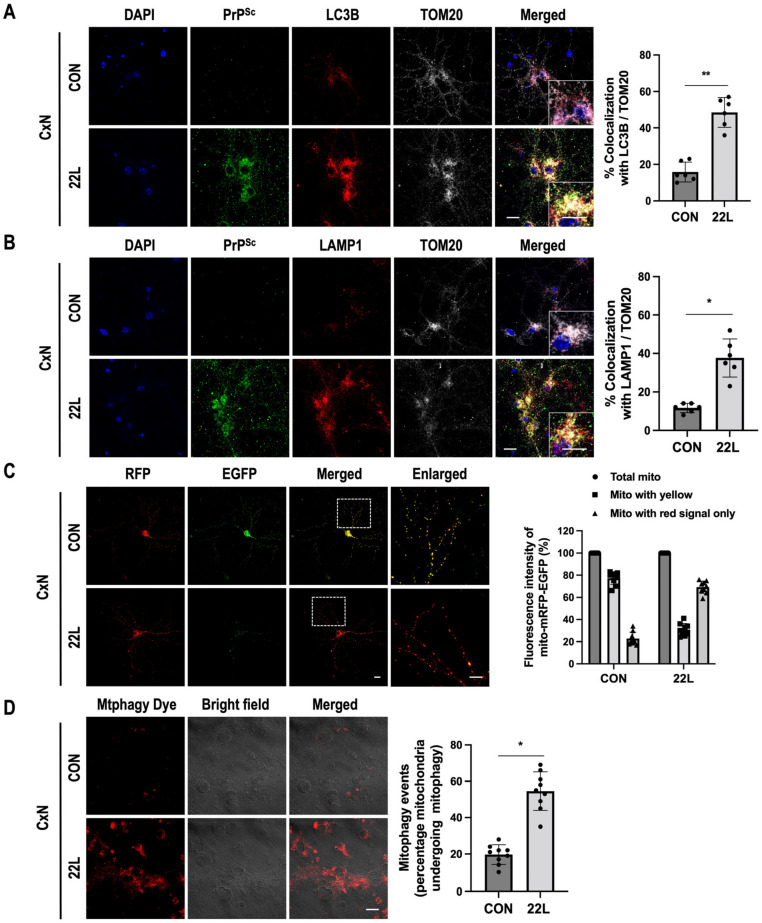
Scrapie infection induces autophagy in mitochondria containing PrP^Sc^. (**A**,**B**) The colocalization of TOM20 (white) with LC3B (red) and LAMP1 (red) in control (CON) or 22L scrapie-infected (22L) CxN cells at 21 dpi was determined using confocal microscopy. Quantification of mitophagosome (**A**) and mitophagolysosome (**B**) formation was analyzed with EzColocalization plugin for ImageJ software version 1.53a (*n* = 6, ** *p* < 0.01, * *p* < 0.05; two-sided Student’s *t*-test). Enlarged insets (white line boxes) showed the sections at a higher magnification. For PrP^Sc^ staining, CxN cells were treated with 98% formic acid for 7 min and then immunostained with an anti-PrP (C-20) antibody (green). (**C**) Transient expression of p-mito-RFP-EGFP by primary cortical neuronal cells targeting mitochondria was determined using confocal microscopy, and mitophagic flux was analyzed with ImageJ software version1.53a (yellow signal, no mitophagy; red signal, mitophagy). DAPI (blue) was used to counterstain the nuclei; enlarged insets (dashed white line boxes) showed the sections at a higher magnification, scale bar, 20 μm. (**D**) Detection of mitophagy with Mtphagy Dye staining (red) using confocal microscopy in control (CON) or 22L scrapie infected (22L) CxN cells at 21 dpi. The intensities of the Mtphagy dye fluorescence were analyzed using ImageJ software and quantified for each group (*n* = 9, * *p* < 0.05; two-sided Student’s *t*-test), *n* = number of independent experiments, scale bar, 20 μm.

A recent study reported that PINK1/Parkin-dependent mitophagy was induced in cultured cells and the brains of experimental mice after prion infection [46]. To examine mitophagy activity in 22L scrapie-infected CxN cells, mitophagosome and mitophagolysosome formation was monitored by immunofluorescence analysis using LC3B and lysosomal-associated membrane protein 1 (LAMP1) as markers for mitophagosomes and mitophagolysosomes, respectively. In scrapie-infected CxN cells, we found excessive induction of mitophagosome and mitophagolysosome formation, and PrP^Sc^ was primarily localized to these structures in the mitochondria (Figure 3A,B). Next, we monitored mitophagic flux using a dual fluorescence p-mito-mRFP-EGFP reporter (pAT016) as described previously [31]. The GFP signal is quenched at lower pH, while RFP can be visualized in both mitophagosomes and acidic mitophagolysosomes; thus, the prevalence of RFP fluorescence in lysosomes indicates the completion of the mitophagic process [31]. As shown in Figure 3C, we observed predominant RFP signals without GFP signals in scrapie-infected CxN cells but not in control cells. This result indicated that scrapie infection induced excessive mitophagy. Additionally, we determined mitophagy in CxN cells using Mtphagy dye staining, which is a quantitative method for evaluating intracellular fluorescence intensity. In correlation with Figure 3C, we confirmed significantly increased mitophagy in 22L scrapie-infected CxN cells compared to control cells (Figure 3D). To further confirm these results, we examined the effect of scrapie infection on the formation of mitophagosomes and mitophagolysosomes in the brains of 22L scrapie-infected mice. As expected, we observed excessive induction of mitophagosome and mitophagolysosome formation in the brains of 22L scrapie-infected mice compared to controls (Supplementary Figure S4A,B). Taken together, our findings suggest that scrapie infection promotes mitophagy by inducing mitophagosome and mitophagolysosome formation.

### 3.4. Scrapie Infection Impairs Mitochondrial Oxidative Phosphorylation (OXPHS) and Alters Glycolytic Metabolism

**Figure 4:**
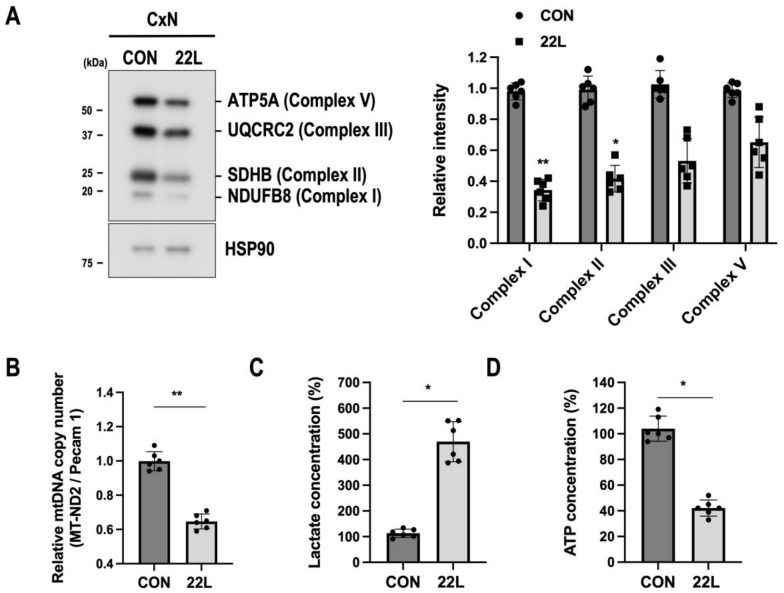
Scrapie infection induces mitochondrial dysfunction associated with defects in OXPHOS-mediated glycolytic metabolism. (**A**) Expression levels of OXPHOS proteins (NDUFB8, SDHB, UQCRC2, ATP5A) in control (CON) and 22L scrapie-infected CxN (22L) cells at 21 dpi were evaluated by Western blot analysis. The intensities of the bands were measured and quantified for each group (*n* = 6, ** *p* < 0.01, * *p* < 0.05; two-sided Student’s *t*-test). (**B**) Quantification of mtDNA copy number by qRT-PCR analysis (*n* = 6, ** *p* < 0.01; two-sided Student’s *t*-test). (**C**,**D**) Relative levels of lactate and ATP in control (CON) or 22L scrapie-infected (22L) CxN cells at 21 dpi were evaluated using a Lactate-Glo assay kit and ATP EnzyLight assay kit with a fluorescence reader (*n* = 6, * *p* < 0.05; two-sided Student’s *t*-test), *n* = number of independent experiments.

Mitochondrial damage, including mitochondrial fragmentation and excessive mitophagy, has a drastic effect on ATP production via the OXPHOS system and is characterized by reduced mtDNA levels in various neurodegenerative diseases [47,48,49]. Therefore, we further investigated the effect of 22L scrapie infection on the OXPHOS system. We found reduced protein levels of mitochondrial complexes I, II, III, and V along with mtDNA depletion in 22L scrapie-infected CxN cells (Figure 4A,B). PrP^C^ is known to be involved in the regulation of metabolic pathways, such as glycolysis, and corruption of PrP^C^ functions by PrP^Sc^ to compromise neuronal metabolism, causing ATP depletion and a shift towards increased glycolysis [50,51]. Thus, we examined whether 22L scrapie infection regulates glycolysis and ATP production in CxN cells. As expected, we observed an increase in lactate concentration (Figure 4C) and a significant reduction in ATP production (Figure 4D) in 22L scrapie-infected CxN cells. Taken together, these results suggest that scrapie infection reduces OXPHOS system-related proteins and mtDNA, thereby affecting its glycolytic pathway and ATP production, which impairs mitochondrial quality control processes followed by increased mitochondrial damage.

### 3.5. Scrapie Infection Induces Apoptosis through Mitochondrial Dysfunction

**Figure 5:**
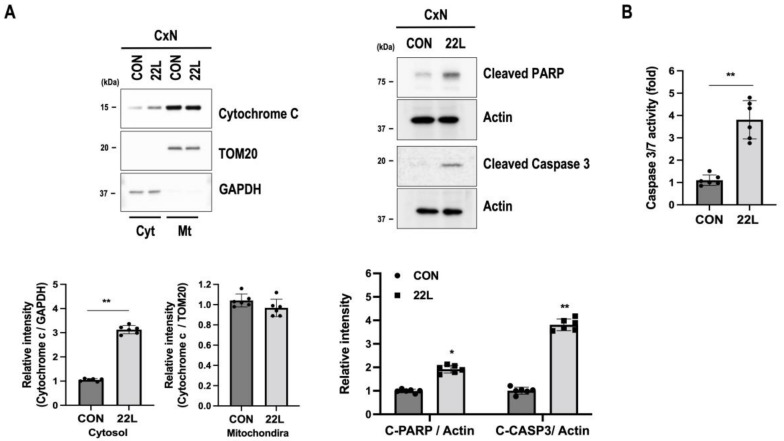
Scrapie infection induces cell apoptosis by promoting caspase 3 activation. (**A**) Expression levels of apoptosis-associated proteins (cytochrome c, cleaved PARP, and cleaved caspase-3) in control (CON) and 22L scrapie-infected (22L) CxN cells at 21 dpi were evaluated with Western blotting. The intensities of the bands were measured and quantified for each group (*n* = 6, ** *p* < 0.01, * *p* < 0.05; two-sided Student’s *t*-test). (**B**) Caspase 3/7 activity was determined with a caspase 3/7 activity assay kit using a fluorescence reader (*n* = 6, ** *p* < 0.01; two-sided Student’s *t*-test), *n* = number of independent experiments. C-PARP, cleaved PARP; C-CASP3, cleaved caspase-3.

Mitochondrial quality control processes are associated with the regulation of apoptosis signaling pathways, including robust cytochrome c release from mitochondria in neurodegenerative diseases [52]. Since we observed impaired mitochondrial quality control in 22L scrapie-infected CxN cells, we investigated the changes in cell-death signaling molecules under scrapie infection by analyzing the release of cytochrome c and the cleavages of PARP and caspase-3. We observed that scrapie infection increased the release of cytochrome c from mitochondria and induced cleavages of PARP and caspase-3 (Figure 5A). We also found that caspase 3/7 enzyme activity was significantly increased in 22L scrapie-infected CxN cells compared to control cells (Figure 5B). These data suggest that scrapie infection-induced mitochondrial dysfunction is involved in apoptotic cell death through the caspase-activation-signaling pathway.

### 3.6. Mitochondrial Quality Control Is Impaired in sCJD Brains

**Figure 6:**
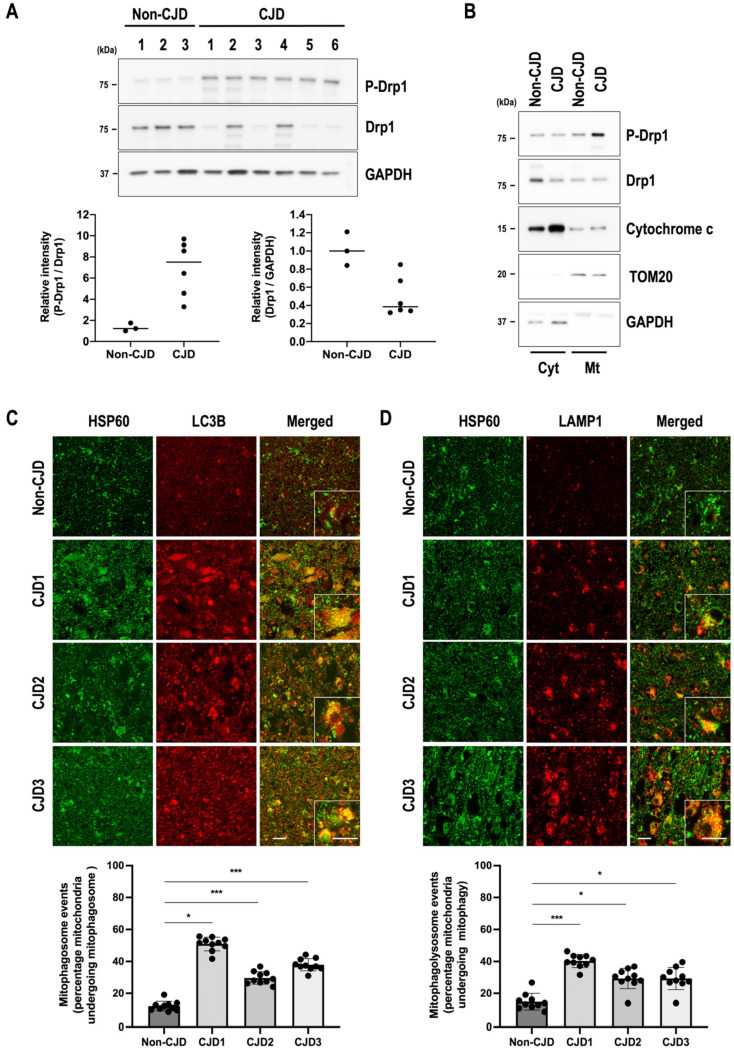
Prion disease induces neuropathogenesis via mitochondrial quality control dysfunction. (**A**) Expression levels of P-Drp1 and total Drp1 in the frontal lobe of non-CJD and sCJD patients were evaluated using Western blotting. (**B**) Mitochondrial localization of P-Drp1 in the cytosolic (Cyt) and mitochondrial (Mt) fractions was analyzed by Western blot analysis. TOM20 and GAPDH were used as markers of the mitochondrial and cytosolic fractions, respectively. (**C**,**D**) The colocalization of HSP60 (green) with LC3B (red) and LAMP1 was determined using confocal microscopy. Quantification of mitophagosome (**C**) and mitophagolysosome (**D**) formation was analyzed using EzColocalization plugin for ImageJ software version 1.53a (*n* = 9 random fields from non-CJD and 3 sCJD patients; *** *p* < 0.001, * *p* < 0.05; one-way ANOVA with Tukey’s post hoc test), *n* = number of random selected fields from 3 CJD patients or 1 non-CJD control. Enlarged inset (white line boxes) showed the sections at a higher magnification, scale bar, 20 μm. Bullets in bar graph represent date set from individual patients.

To confirm the alterations in mitochondrial quality control in the frontal lobe of sCJD patients, Drp1 phosphorylation and excessive mitophagy were examined by Western blot analysis and immunofluorescence staining. As shown in Figure 6A, interestingly, the mitochondrial fission-associated protein Drp1 was highly phosphorylated in all sCJD patients, whereas the expression of the total form of Drp1 tended to be lower than that in non-CJD patients. Additionally, the level of P-Drp1 was predominantly increased in the mitochondrial fractions of the brains of sCJD patients and promoted the release of cytochrome c from mitochondria (Figure 6B). We also performed double immunofluorescence staining to investigate the formation of mitophagosomes and mitophagolysosomes (mitophagy process) in the frontal lobe of non-CJD and sCJD patients. We found that localization of the mitophagosome marker LC3B or the lysosomal marker LAMP1 was significantly increased in the mitochondrial HSP60-positive cells of the frontal lobe of sCJD patients compared with that of non-CJD patients (Figure 6C,D). These results suggest that mitochondrial quality control defects may lead to neurodegeneration and pathological changes in prion diseases.

## 4. Discussion

**Figure 7:**
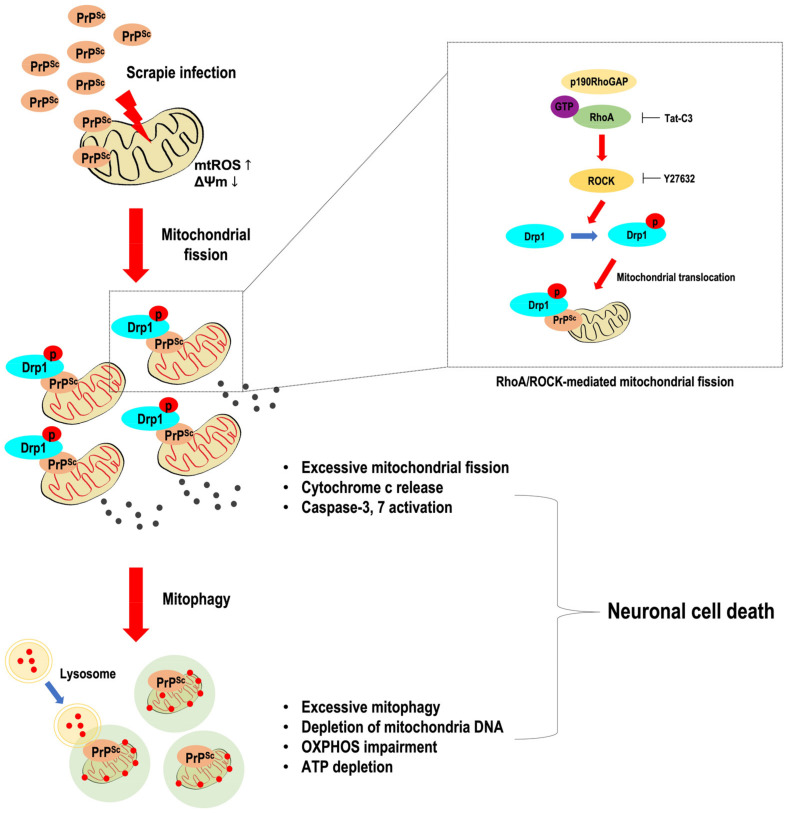
Schematic representation of PrP^Sc^-mediated neuronal cell death through mitochondrial dysfunction. Scrapie infection increased the phosphorylation of Drp1 at serine 616 in the mitochondria via RhoA/ROCK activity, leading to the activation of mitochondrial fission, accompanied by excessive mitophagy. Moreover, scrapie infection also increased cytochrome c release from the mitochondria and reduced mitochondrial function. Subsequently, excessive mitochondrial fission and mitophagy resulted in enhanced caspase 3 activity and PARP cleavage and hampered ATP production by impairing OXPHOS, facilitating neuronal cell death.

Mitochondrial quality control dysfunction results in the accumulation of defective mitochondria, which leads to cell death and dysfunction in neurodegenerative diseases such as AD, PD, HD, and prion diseases [53]. The present study demonstrated abnormal mitochondrial quality control in prion disease. This dysfunction was due to increased levels of activated mitochondrial fission proteins and excessive mitophagy, which resulted in the excessive removal of mitochondria and impairment of cellular energy metabolism followed by neuropathological changes in prion diseases (Figure 7).

Mitochondrial fission is critical for the regulation of mitophagy and mitochondrial quality control, which are associated with neurodegeneration. Under normal conditions, mitochondrial quality control is an excellent protective response to various stressors in the cell [54,55]. Moreover, aberrations in the cellular mitochondrial fission pathway are linked to various pathophysiological conditions, increased mtROS generation, and aggregated protein accumulation [17,49,50]. These mechanisms are involved in supplying oxygen and energy sources for cell survival and triggering mitochondrial fragmentation. Furthermore, these key processes are associated with mtROS-induced neuronal cell death, which facilitates the neuropathogenesis and progression of neurodegenerative diseases [56,57]. The generation of mtROS is activated through various signaling pathways, such as the RhoA-mediated pathway, which is activated in response to oxidative stress and leads to excessive mitochondrial fission accompanied by increased mitochondrial autophagy (mitophagy) and decreased ATP production and contributes to cell death [38,53,54].

PrP^C^ is involved in the regulation of apoptosis, likely by altering mitochondrial Ca^2+^ concentrations and increasing mtROS, leading to ΔΨm loss and cytochrome c release [58,59,60]. Moreover, the PrP 106–126 peptide, which shares several characteristics with PrP^Sc^, also induces mitochondrial dysfunction via mtROS in in vitro models [61,62]. These findings suggest that increased mtROS during scrapie infection augments aberrant mitochondrial fission, which may be related to the progression of the pathological changes that are observed in prion disease.

Mitochondrial fission is regulated by the phosphorylation of Drp1 at serine 616 (P-Drp1), which is mediated by a variety of protein kinases (i.e., CDK1/cyclin B, CaMKII, and RhoA/ROCK) [54,60,61]. Activation of these protein kinases induces mitochondrial fission and mitophagy. Previous studies have also reported that scrapie infection induces the activation of the RhoA/ROCK signaling pathway and is associated with prion pathogenesis [44]. Furthermore, RhoA/ROCK activity regulates mitochondrial fission by promoting the P-Drp1 and its translocation to mitochondria [45]. We found significantly increased P-Drp1 in mitochondria and enhanced mitochondrial fission in scrapie-infected CxN cells as well as prion-affected in vivo models. In addition, the inhibition of ROCK and RhoA using Y27632 and Tat-C3, respectively, reduced mtROS and partially restored ΔΨm, which gives rise to the possibility that scrapie infection exerts its effect on mitochondrial fission via modulation of RhoA-mediated signaling effectors.

Excessive mitochondrial fission is associated with mitochondrial dysfunction via mitophagy. In addition, aberrant mitophagy induces cell death due to an OXPHOS system deficiency and consequent energy deficiency, which is involved in neuropathogenesis [63,64,65]. Mitophagy regulates the degradation of defective mitochondria [66]. However, various pathophysiological conditions, such as neurodegeneration, cancer, and ageing, cause the overactivation of mitophagy, resulting in the excessive elimination of mitochondria [67,68]. The reduction in the mitochondrial population mediates the impairment of energy metabolism due to OXPHOS system defects, which exacerbates neuronal cell death. In addition, energy failure, such as OXPHOS system deficiency, can be triggered by an imbalance in mitochondrial turnover, such as excessive mitochondrial removal, particularly in the mitochondrial quality control process. Moreover, an OXPHOS system deficiency leads to enhanced glycolysis, which increases the levels of oxidized glutathione and increases susceptibility to ROS formation in cells [69]. These OXPHOS system deficiencies induce excessive lactate production, resulting in elevated lactate levels in the cerebrospinal fluid (CSF) of patients with neurodegenerative diseases [70,71], and are involved in modulating several immune-related processes, such as inflammation [72]. Therefore, OXPHOS system deficiencies could occur during neuronal cell death throughout disease progression, and the malfunction of mitochondrial quality control, which induces a variety of metabolic changes, could lead to the failure to mount an efficient inflammatory immune response. This scenario is supported by our findings on mitochondrial quality control malfunctions and the abovementioned observations on the relationships between mitochondrial quality control and scrapie-induced neuropathological changes. Because current knowledge of the molecular features of mitochondrial quality control is limited, it remains unclear whether mitochondrial quality control, such as the mitophagy-signaling pathway, plays a role in the onset and/or progression of prion neuropathogenesis.

In conclusion, our findings suggest that impaired mitochondrial quality control by abnormal mitochondrial fission and mitophagy after scrapie infection may play an important role in prion neuropathogenesis. Further studies are necessary to fully elucidate the molecular mechanisms of mitochondrial quality control under physiological conditions in healthy and diseased states and to provide mitochondria-target potential therapeutic approaches aimed at the maintenance of normal mitochondrial quality control to mitigate neuropathogenesis in prion diseases.

## Figures and Tables

**Table 1:** Characterization of human subjects.

Subject	Diagnosis	Sex	Age	Brain Weight (g)	PrP^Sc^ Type	PMD (Hours)	Applications
Control 1	Non-CJD	M	83	1220	ND	12	WB
Control 2	Non-CJD	M	86	1100	ND	2.5	WB
Control 3	Non-CJD	M	71	1225	ND	7	WB
^#^ Control 4	Non-CJD	F	70	1300	ND	12	ICC
CJD 1	Sporadic CJD	M	77	1600	1	2.5	WB
CJD 2	Sporadic CJD	M	66	1380	1	13	WB, ICC
CJD 3	Sporadic CJD	F	65	1170	1	24	WB
CJD 4	Sporadic CJD	M	49	1150	1 and 2	120	WB
CJD 5	Sporadic CJD	M	50	1550	1	12	WB
CJD 6	Sporadic CJD	M	58	1040	1	12	WB
CJD 7	Sporadic CJD	M	58	1250	1	12	ICC
CJD 8	Sporadic CJD	M	66	1510	1	24	ICC

PMD, Post-mortem interval. ND, Not detected. Control specimens were obtained from individuals without CJD and AD. ^#^ Control 4, viral encephalitis. WB, Western blotting; IHC, immunohistochemistry; ICC, immunocytochemistry.

**Table 2:** Primers for qRT-PCR.

Gene	Sequence (Forward/Reverse)	Product Length (bp)
Drp1	5′-CGTGACAAATGAAATGGTGC-3′ 5′-CATTAGCCCACAG-GCATCAG-3′	216
Actin	5′-GACCTCTATGCCAACACAGT-3′ 5′-AGTACTTGCGC-TCAGGAGGA-3′	139
mtDNA (MT-ND2, NADH dehydrogenase)	5′-CCTATCACCCTTGCCATCAT-3′ 5′-GAGGCT GTTGCTTGTGTGAC-3‘	193
nucDNA (Pecam 1)	5′-ATGGAAAGCCTGCCATCATG-3′ 5′-TCCT TGTTGTTCAGCATCAC-3′	235

## Data Availability

The data presented in this study can be obtained upon a reasonable request to corresponding author (E.-K.C).

## Supplementary Materials

The following supporting information can be downloaded at: https://www.mdpi.com/article/10.3390/cells11172744/s1, Figure S1. Scrapie infection induces Drp1-mediated mitochondrial fission in the brain of 22L scrapie-infected mice; Figure S2. RhoA/ROCK activity is involved in Drp1-mediated mitochondrial fission in 22L scrapie-infected CxN cells; Figure S3. RhoA/ROCK activity is involved in mtROS generation and ΔΨm in 22L scrapie-infected CxN cells; Figure S4 Scrapie infection induced mitophagy in the brains of 22L scrapie-infected mice.
