# Supplementary material for: Impairment of Neuronal Mitochondrial Quality Control in Prion-Induced Neurodegeneration
Source: Cells. 2022 Sep 2;11(17):2744. doi: 10.3390/cells11172744 (PMC9454542; doi:10.3390/cells11172744)
Supplement: Supplementary file 1 [file cells-11-02744-s001.zip › cells-1826428-supplementary.pdf]

Supplementary Material

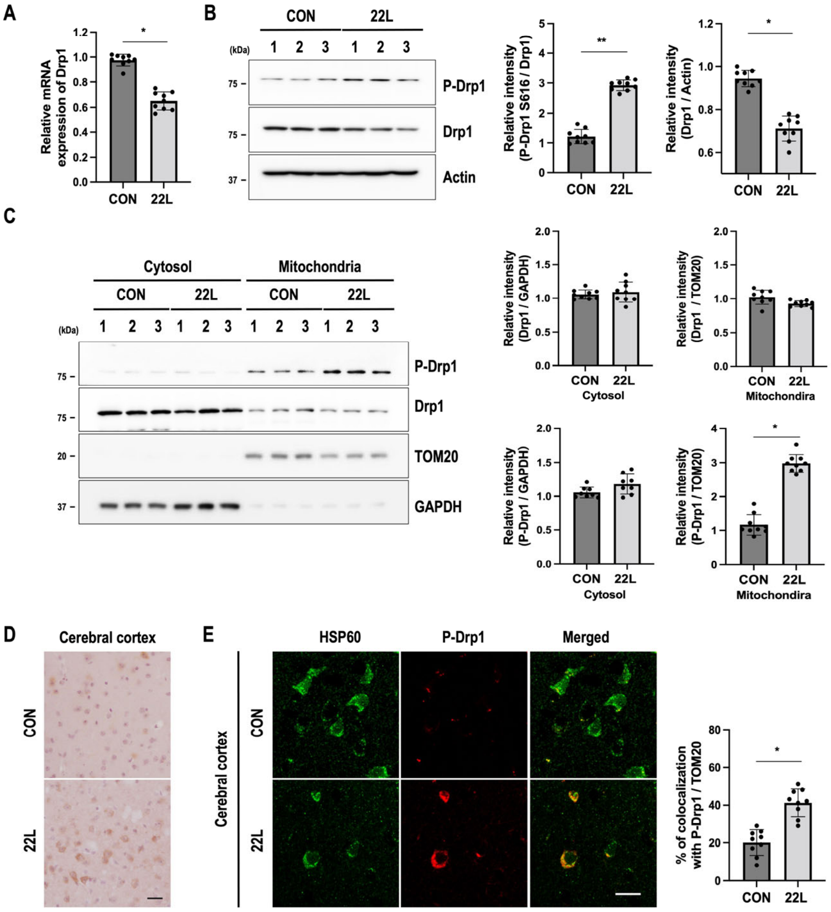

Figure S1. Scrapie infection induces Drp1-mediated mitochondrial fission in the brain of 22L scrapie-infected mice. (**A**) The mRNA level of Drp1 was analysed using qRT-PCR (n = 9, * $p < 0.05$). (**B**) The P-Drp1 in the brains of 22L scrapie-infected mice was examined by Western blotting. The band intensities for each group were measured and quantified (n = 9, ** $p < 0.01$, * $p < 0.05$). (**C**) Drp1 and P-Drp1 levels in the cytosolic and mitochondrial fraction were detected by Western blotting. GAPDH and TOM20 were used as markers of the cytosolic and mitochondrial fractions, respectively. The band intensities for each group were measured and quantified (n = 9, * $p < 0.05$). (**D**) Immunohistochemical staining of P-Drp1 in the cerebral cortex of 22L scrapie-infected mice. (**E**) Colocalization of HSP60 (green) and P-Drp1 (red) in the cerebral cortex of 22L scrapie-infected mice was analysed using EzColocalization plugin for ImageJ software version 1.53a (n = 9, * $p < 0.05$), n = total number of mice (**A**–**C**) or random selected fields (**E**), scale bar, 20 μm. Data were quantified from three independent experiments.

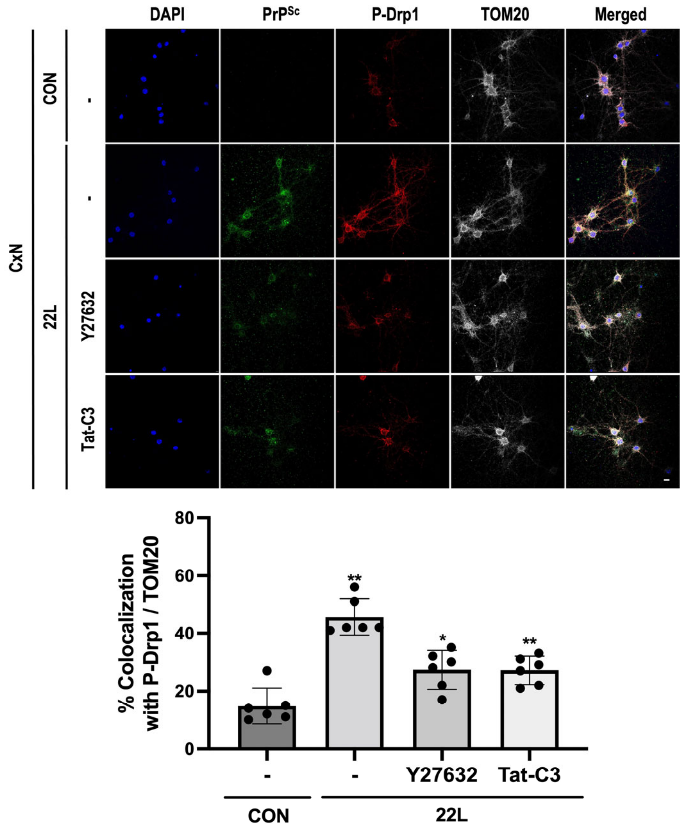

Figure S2. RhoA/ROCK activity is involved in Drp1-mediated mitochondrial fission in 22L scrapie-infected CxN cells. The control (CON) or 22L scrapie-infected (22L) CxN cells were incubated with or without 20 μM Y27632 or 1 μg/mL Tat-C3 at 17 dpi for 4 days. (A) The triple-localization of PrP^{Sc} (green) with P-Drp1 (red) and TOM20 (white) was determined using confocal microscopy. Mitochondrial fission was quantified by detecting P-Drp1 and TOM20, and mitochondrial translocation was analysed using EzColocalization plugin for ImageJ software version 1.53a (n = 6, ** $p < 0.01$, * $p < 0.05$; one -way ANOVA with Tukey's post hoc test). DAPI (blue) was used to counterstain the nuclei; n = number of independent experiments, scale bar, 20 μm.

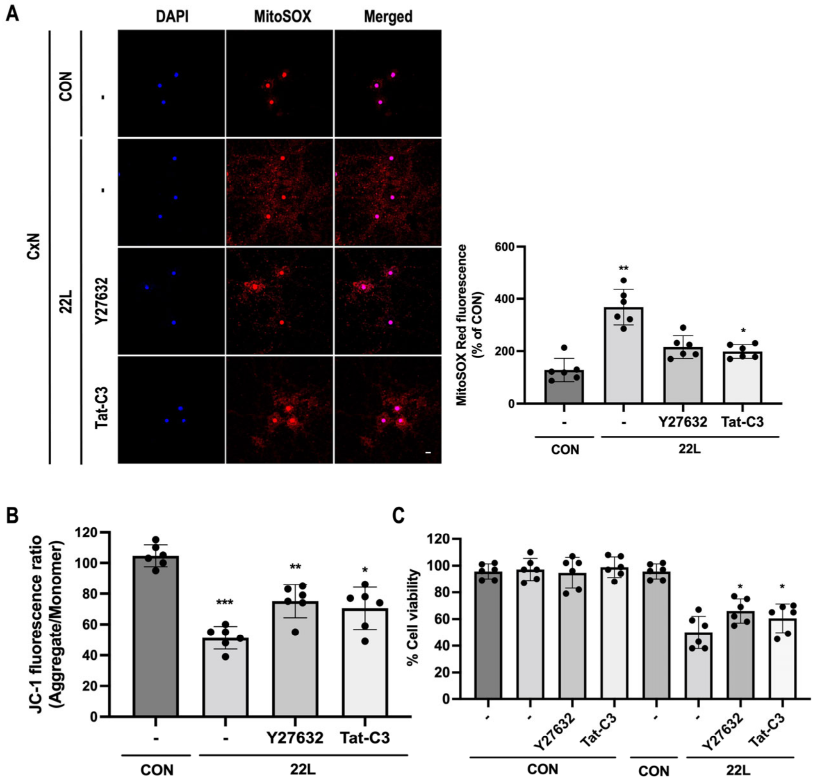

Figure S3. RhoA/ROCK activity is involved in mtROS generation and ΔΨm in 22L scrapie-infected CxN cells. The control (CON) or 22L scrapie-infected (22L) CxN cells were incubated with or without 20 µM Y27632 or 1 µg/mL Tat-C3 a t 17 dpi for 4 days. (**A**) mtROS were detected using MitoSOX staining (red) and confocal microscopy. The mtROS fluorescence intensities for each group were measured and quantified (n = 6, ** $p < 0.01$, * $p < 0.05$; one-way ANOVA with T ukey's post hoc test). DAPI (blue) was used to counterstain the nuclei; scale bar, 20 µm. (**B**) The ΔΨm was evaluated using JC−1 staining and a fluorescence reader (n = 6, *** $p < 0.001$, ** $p < 0.01$, * $p < 0.05$; one-way ANOVA with Tukey's pos t hoc test). (**C**) The relative viability of control (CON) or 22L scrapie-infected (22L) CxN cells treated with or without 20 µM Y27632 or 1 µg/mL Tat-C3 was evaluated using a CCK8 assay kit and a fluorescence reader (n = 6, * $p < 0.05$; one-way ANOVA with Tukey's post hoc test), n = number of independent experiments.

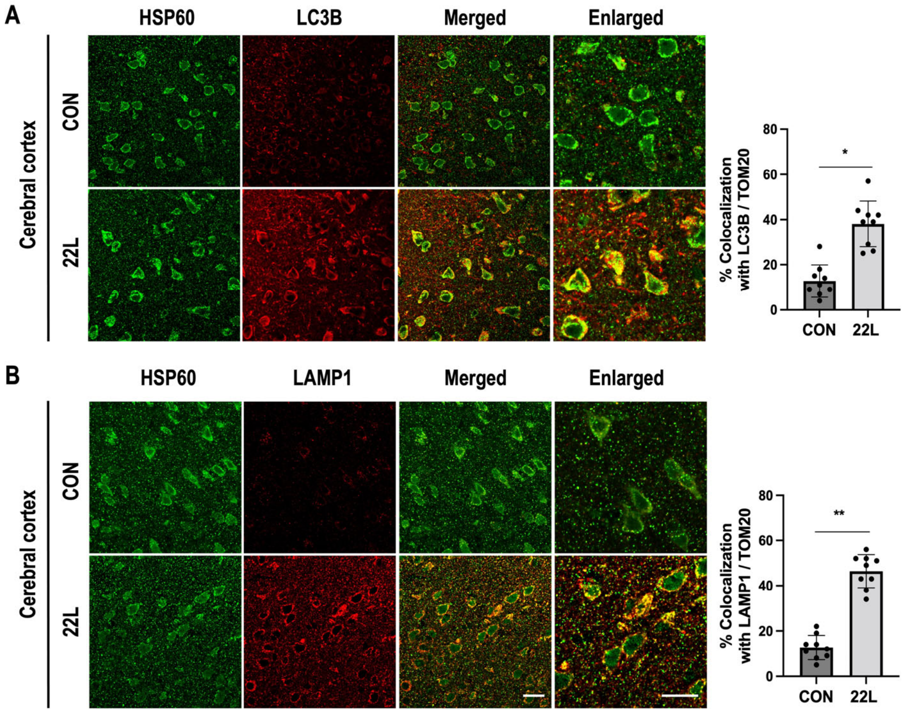

Figure S4. Scrapie infection induced mitophagy in the brains of 22L scrapie-infected mice. The colocalization of HSP60 (green) with LC3B (red) and LAMP1 (red) was determined using confocal microscopy. Mitophagosome (**A**) and mitop hagolysosome (**B**) formation was quantified and analysed using ImageJ software version 1.53a (n = 9 random fields from 3 mice per group; ** $p < 0.01$, * $p < 0.05$; two-sided student's t-test). Scale bar, 20 μm.
